# Mechanosensory Signaling in Enterochromaffin Cells and 5-HT Release: Potential Implications for Gut Inflammation

**DOI:** 10.3389/fnins.2016.00564

**Published:** 2016-12-19

**Authors:** Andromeda Linan-Rico, Fernando Ochoa-Cortes, Arthur Beyder, Suren Soghomonyan, Alix Zuleta-Alarcon, Vincenzo Coppola, Fievos L. Christofi

**Affiliations:** ^1^Department of Anesthesiology, Wexner Medical Center at Ohio State UniversityColumbus, OH, USA; ^2^CONACYT-Centro Universitario de Investigaciones Biomedicas, University of ColimaColima, Mexico; ^3^Enteric Neuroscience Program, Division of Gastroenterology and Hepatology, Department of Physiology and Biomedical Engineering, Mayo ClinicRochester, MN, USA; ^4^SBS-Cancer Biology and Genetics, Ohio State UniversityColumbus, OH, USA

**Keywords:** mechanotransduction, enterochromaffin, Piezo 2, purinergic receptors, ENS, inflammation

## Abstract

Enterochromaffin (EC) cells synthesize 95% of the body 5-HT and release 5-HT in response to mechanical or chemical stimulation. EC cell 5-HT has physiological effects on gut motility, secretion and visceral sensation. Abnormal regulation of 5-HT occurs in gastrointestinal disorders and Inflammatory Bowel Diseases (IBD) where 5-HT may represent a key player in the pathogenesis of intestinal inflammation. The focus of this review is on mechanism(s) involved in EC cell “mechanosensation” and critical gaps in our knowledge for future research. Much of our knowledge and concepts are from a human BON cell model of EC, although more recent work has included other cell lines, native EC cells from mouse and human and intact mucosa. EC cells are “mechanosensors” that respond to physical forces generated during peristaltic activity by translating the mechanical stimulus (MS) into an intracellular biochemical response leading to 5-HT and ATP release. The emerging picture of mechanosensation includes Piezo 2 channels, caveolin-rich microdomains, and tight regulation of 5-HT release by purines. The “*purinergic hypothesis”* is that MS releases purines to act in an autocrine/paracrine manner to activate excitatory (P2Y_1_, P2Y_4_, P2Y_6_, and A_2A_/A_2B_) or inhibitory (P2Y_12_, A_1_, and A_3_) receptors to regulate 5-HT release. MS activates a P2Y_1_/G_α_q/PLC/IP_3_-IP_3_R/SERCA Ca^2+^signaling pathway, an A_2A_/A_2B_–Gs/AC/cAMP-PKA signaling pathway, an ATP-gated P2X_3_ channel, and an inhibitory P2Y_12_-G_i/o_/AC-cAMP pathway. In human IBD, P2X_3_ is down regulated and A_2B_ is up regulated in EC cells, but the pathophysiological consequences of abnormal mechanosensory or purinergic 5-HT signaling remain unknown. EC cell mechanosensation remains poorly understood.

## Introduction

The enterochromaffin cell (EC) synthesizes and releases 5-hydroxytryptamine (5-HT), which is involved in mucosal secretory reflexes, motility and transmission of information about visceral pain sensation (Cooke and Christofi, 2006; Christofi, 2008; Mawe and Hoffman, 2013). The available evidence suggests that alterations in 5-HT regulation and signaling mechanisms (e.g., *5-HT secretion, availability, and re-uptake mechanisms*) may contribute to the pathogenesis of inflammatory bowel diseases (IBD), irritable bowel syndrome (IBS, including post-infectious IBS), carcinoid syndrome, vomiting and diarrhea after radiotherapy or platinum chemotherapy, and diarrhea associated with bacterial toxin induced enterocolitis (Fujimiya et al., 1997; Linden et al., 2003; Coates et al., 2004; Crowell, 2004; Crowell et al., 2004; Galligan, 2004; Gershon, 2004; Kordasti et al., 2004; O'Hara et al., 2004; Linan-Rico et al., 2013a). Possible associations also exist with celiac disease, diverticular disease and colorectal cancer (Manocha and Khan, 2012). Our challenge is to better understand how EC cells regulate 5-HT release before we can truly appreciate the consequences of abnormal 5-HT signaling in diseases.

EC cells function as chemical and mechanical transducers. EC cells act as chemosensors by detecting changes in the chemical milieu of the luminal environment of the gut and respond to nutrients (such as free fatty acids, monosaccharides, peptides, amino acids), purines such as adenosine and ATP, the concentration of the solute or changes in pH to alkaline or acidic—chyme (Kim et al., 2001a; Cooke et al., 2003). For example, human EC cells can act as glucose sensors during ingestion of a meal and respond by secreting 5-HT (Kim et al., 2001b). Gut EC cells can also act as oxygen sensors (Haugen et al., 2012). In addition, EC cells are sensory detectors of mechanical forces operating during intestinal peristalsis, i.e., by acting as “mechanosensors.” Various mechanical forces lead to 5-HT release to initiate or contribute to gut neural reflexes that coordinate motility and secretion, peristaltic waves, mixing movements (fed state), the migrating motor complex (fasted state), and mass movement during the defecation reflex. During such complex motor behaviors, mechanical forces that may trigger 5-HT release under physiological conditions include tensile force and flow shear stress, intraluminal pressure, turbulent and centrifugal forces, stretch/distension, touch, compression, membrane distortion/deformation and changes in cell volume.

**Figure 1:**
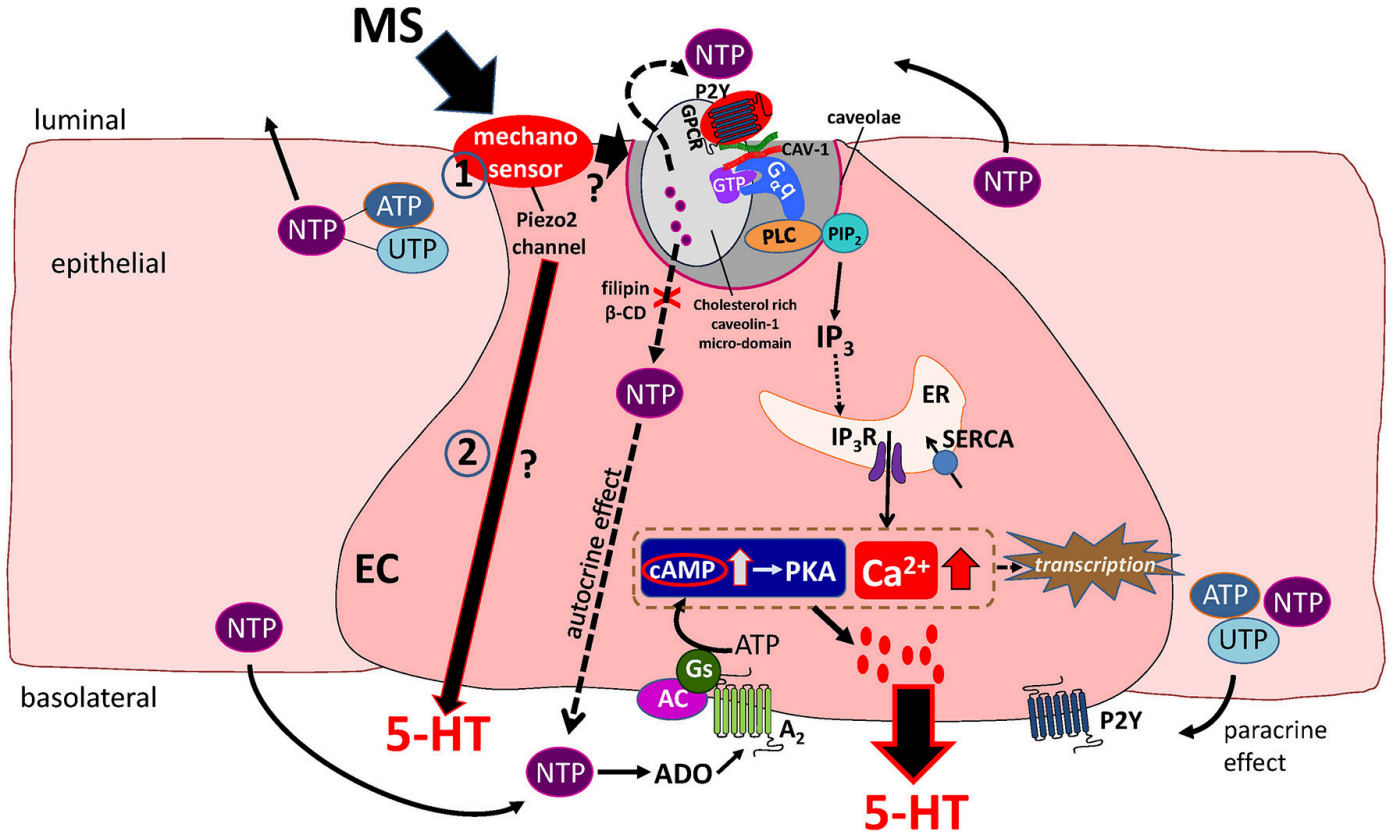
**Working Hypothesis of mechanotransduction in EC cells**. Mechanical stress (MS) activates a mechanosensor in EC and epithelial cells to induce release of 5′nucleotide triphosphates (NTP) such as ATP and UTP that act in an autocrine or paracrine manner to modulate 5-HT release. The Piezo 2 mechanogated channel was recently identified as a critical component of the mechanosensor and mechanotransduction signaling pathway activated by MS in EC cells leading to 5-HT release (in pathways 1 and 2 in the diagram). Mechanically evoked NTP release activates a predominant P2Y_1_/G_α_q/PLC/PIP_2_/IP_3_/IP_3_R/SERCA pump–Ca^2+^signaling pathway leading to 5-HT release. Caveolin-1 (CAV-1) associated with cholesterol-rich micro-domains in caveolae (*specialized invaginations in the lipid bilayer of the EC cell membrane*) forms a scaffold to support the functional coupling of the P2Y_1_-GPCR, Gαq, PLC and NTP secretion from the cell. In this model, caveolae and cholesterol rich caveolin-1 microdomains are essential for both NTP release and down-stream Ca^2+^dependent 5-HT release. Therefore, manipulations that disrupt the structure or assembly of caveolae by treating cells to filipin or β-cyclodextrin (β-CD) prevent the mechanically evoked NTP (ATP) and 5-HT secretion. A minor mechanosensitive pathway is an A_2_/Gs/AC-cAMP/PKA signaling pathway of 5-HT release. Ca^2+^ and cAMP-dependent transcriptional regulation occurs in response to mechanical stimulation that can further modulate EC cell function(s). In this model, it is postulated that Piezo 2 activation could also stimulate 5-HT secretion via a separate purine-independent pathway (pathway 2 in diagram).

**Figure 2:**
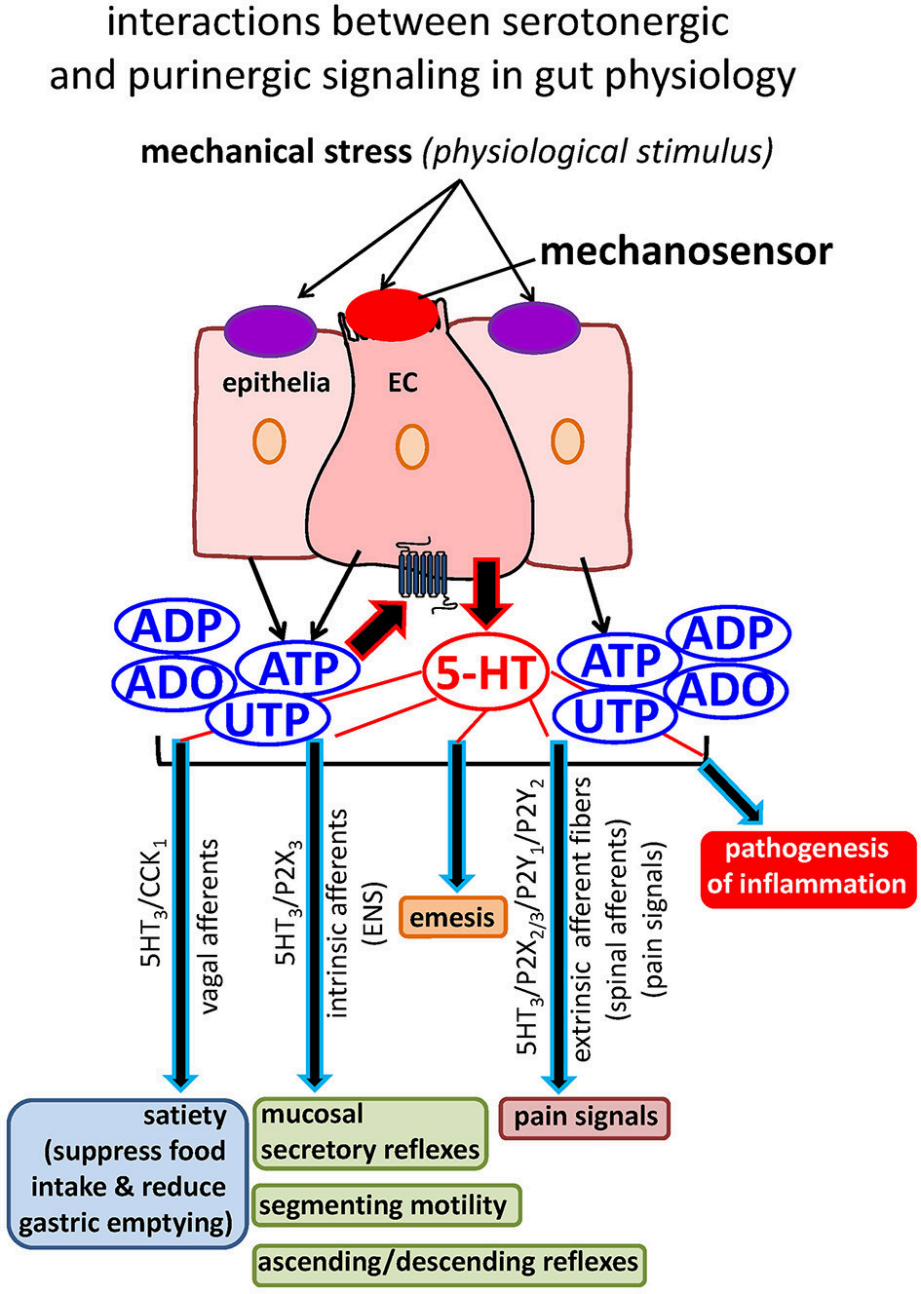
**Interactions between serotonergic and purinergic signaling in gut physiology**. Mechanical stress activates a mechanosensor on EC to release 5-HT that has a myriad of physiological functions, including activation of enteric neural secretory and motility reflexes, transmission of satiety signals, transmission of pain signals, and induction of emesis. Mechanical stress also releases purinergic mediators (ATP and UTP) from EC and epithelial cells to tightly modulate the secretion of 5-HT as well as gut reflexes. Abnormal 5-HT signaling occurs in GI disorders and IBD, but little is known about how this occurs in EC cells. 5-HT is also implicated in the pathogenesis of inflammation. Purinergic signaling is very sensitive to inflammation, and this is also the case in EC cells. Our working hypothesis is that purinergic mechanisms are linked to abnormal 5-HT secretion and hence signaling in inflamed gut.

*In vitro* studies on EC cells have explored the impact of mechanical stimulation on 5-HT release, and data in freshly isolated EC cells and EC cell lines have provided important new insights into the mechanosensory signaling pathways. While it is now possible to isolate human EC cells from surgical specimens (Kidd et al., 2006; Raghupathi et al., 2013) or mouse EC cells from CFP expressing Tph1-CFP cells (Li et al., 2014) to study 5-HT release, much of our knowledge comes from studies using the BON cell model. This model has provided significant new insights into mechanisms and processes involved in translating a mechanical stimulus into 5-HT release to trigger gut reflexes. *We forward a unified purinergic hypothesis of mechanosensation for modulation of 5-HT release, gut reflexes, visceral sensation and pain, and summarize the evidence in this review to support it (illustrated in Figures* *1*, *2**)*. Enteric neural regulation of mucosal secretion and purinergic signaling in secretomotor function were previously extensively reviewed (Cooke and Christofi, 2006; Christofi, 2008).

More than a decade ago, we published an article in News in Physiological Sciences titled “*The Force Be with You”: ATP in Gut Mechanosensory Transduction (Cooke et al., 2003)*. The article emphasized a fundamental principle that mechanical stimulation releases nucleotides, ATP and UTP from cells in the body. Mechanical forces generated during peristalsis release nucleotides leading to secretion of the sensory mediator 5-HT from EC cells and act as autocrine, paracrine or neurocrine mediators in neural reflexes regulating chloride secretion. Despite some progress in the field, there are still many unresolved questions: *How do these cells detect “physical forces” due to mechanical activation and convert them to biological responses in the intestine? What is the mechanosensor? What is the mechanotransducer? What are the critical signaling pathways linked to 5-HT release? What is the influence of intestinal inflammation or GI disease on mechanosensation in EC cells?* The focus of this review will be on *mechanotransduction in EC* cells to address some of these questions, with special attention to mechanogated channels, adenosine, ATP, UTP, G protein coupled receptors (GPCRs), the lipid membrane layer and caveolin-1. The precise molecular mechanisms by which EC cells transduce a mechanical stimulus (MS) into the physiological response, 5-HT release, are currently under investigation. Emerging evidence supports a role for abnormal purinergic modulation of 5-HT secretion during intestinal inflammation that could affect a wide variety of physiological responses. Based on our current understanding of purinergic signaling in health, disease and therapeutics (Ochoa-Cortes et al., 2014), *we further propose that purinergic pathways in EC cells are a potential therapeutic target in gastrointestinal (GI) disorders associated with abnormal 5-HT signaling*.

### The human BON cell model

EC cells synthesize 95% of the total body's 5-HT and the release of 5-HT has important physiological effects in the gut. However, to date, there are only a few studies on the cellular and molecular mechanisms of 5-HT release from single primary human EC cells (Kidd et al., 2006; Chin et al., 2012; Raghupathi et al., 2013). Most of our knowledge comes from studies in cell line models of EC cells, including KRJ-1 (Siddique et al., 2009), RIN14B (Nozawa et al., 2009), and in particular BON cells (Kim et al., 2001a). Novel isolation techniques for EC cells from normal or diseased tissues in recent years provide an opportunity to test important hypotheses generated using the BON cell model system. A rapid filtration/isolation technique of human and guinea pig EC cells (without the need for FACS sorting) allows analysis of single EC cell function by monitoring real-time 5-HT release by electrochemical detection, Ca^2+^imaging and patch-clamp recording (Raghupathi et al., 2013).

Parekh et al. (1994) first described the *in vitro* characterization of the human carcinoid BON cell line over 20 year ago. BON cells originated from an operative specimen of the peripancreatic lymph node in a 28 year old man with a metastatic carcinoid tumor of the pancreas. BON cells grow in culture and provide a suitable *in vitro* model to study 5-HT secretion or other mediators in human enterochromaffin cells (EC). Cells in culture express 5-HT, 5-HT transporter (SERT), pancreastatin, neurotensin, chromogranin A (CgA), bombesin, GABA, synaptophysin, and secretogranin II. The cells do not express glial (glial fibrillary acidic protein) or neuronal (neurofilament) markers. Functional receptors exist for acetylcholine, 5-HT, somatostatin (SST_2_), isoproterenol (β-adrenergic), VIP (VPAC1), PACAP, CRF_1_, TRPA1 channels, TRPM8 channels, CRH, CRF, dopamine, bradykinin, immunologics (e.g., IL-13), VMAT2, VGLUT2, adenosine receptors (A_1_, A_2A_, A_2B_, and A_3_), and nucleotide receptors for P2X and P2Y_1_, P2Y_4_, P2Y_6_, and P2Y_12_ receptors. Purinergic receptors for adenosine and nucleotides (ATP, ADP) have been linked to mechanosensory signaling pathways in EC cells (Cooke et al., 2003; Cooke and Christofi, 2006; Christofi, 2008; Linan-Rico et al., 2013a, 2014).

5-HT, 5-hydroxytryptophan (5-HTP), and 5-hydroxyindoleacetic-acid (5-HIAA) are detected by HPLC in BON cells and in the media of cultured cells. Deamination of 5-HT to 5-HIAA is catalyzed by the enzyme monoamine oxidase (MAO) that is present in BON cells. 5-HT receptors are likely to be expressed on BON cells, since 5-HT that is synthesized and secreted by BON cells could stimulate the release of other mediators such as neurotensin and pancreastatin (Feldman, 1989). BON cells possess a specific transport system for the uptake of 5-HT demonstrated by showing that ^3^H-5-HT uptake is inhibited by fluoxetine (Parekh et al., 1994). The transport system is a mechanism for modulation of the biological effects of amines by reducing their local concentration (Bonanno and Raiteri, 1987).

### Similarities and differences between primary EC cells and BON cells

Despite its pancreatic origin, the BON cell line has been the most widely used *in vitro* EC cell model to date. It is therefore, important to briefly highlight some of the similarities and differences between the BON cell line, EC-cell derived cell lines and normal EC cells. Siddique et al. (2009) carried out a comparative analysis between the BON cell line and the small intestine EC-cell derived ileal neuroendocrine tumor cell line KRJ-1 in order to define an appropriate EC cell neuroendocrine tumor model. Pharmacological analysis indicated that isoproterenol, noradrenaline and PACAP could stimulate 5-HT release in both cell lines, but agonists had lower efficacy in BON cells. Somatostatin inhibited 5-HT release with a similar efficacy. On the other hand, acetylcholine and cholecystokinin inhibited release of 5-HT in KRJ-1 cells but stimulated release in BON cells. Molecular analysis revealed substantial differences in gene transcriptome profiles between the two cell lines, and in comparison to normal jejunum. Differences also occurred in receptor expression profiles for muscarinic receptors (M1-M4), TGFβR2 and somatostatin receptors. Substance P, guanylin, CgA and NSE were expressed in both cell lines. Differences in cell origin (i.e., EC-cell derived KRJ-1 vs. pancreatic origin of BON), regional differences, or neoplastic transformation in each cell line can account for differences between the two cell lines.

Tph1, the enzyme involved in 5-HT synthesis is present in normal EC cells and KRJ-1 cells, indicating that 5-HT synthesis is regulated by the Tph1 isoform; the Tph2 isoform is not present in these cells or normal EC cells. In contrast, BON cells express Tph1 and Tph2 transcripts and DOPA decarboxylase, an enzyme implicated in the synthesis of dopamine and 5-HT (Siddique et al., 2009). This may account for some of the variability in 5-HT secretion seen in BON cells placed in culture over-time (i.e., continuous culture for 7 days) and this is an important consideration for release experiments in BON cells.

TRPA1 is a sensor molecule for EC cells (Nozawa et al., 2009). It is highly expressed in EC cells and TRPA1 agonists stimulate EC cell functions including elevating intracellular Ca^2+^ levels and 5-HT release in highly concentrated rat EC cell fractions (i.e., 81% of cells were positively stained for 5-HT) or the RIN14B, a rat pancreatic EC cell line; its role in BON cells, another EC cell model of pancreatic origin remains unknown. Similar to BON cells, *Mastomys* ileal EC cells (Kidd et al., 2006) express VPAC_1_ and somatostatin 2 receptors. Forskolin, isoproterenol and PACAP stimulate 5-HT release. Isoproterenol also stimulates cAMP levels in these cells. Osteotride and GABA_A_ inhibit 5-HT release. These characteristics are shared with BON cells, whereas other responses such as those to acetylcholine and bombesin are different (see reviews by Christofi, 2008; Cooke and Christofi, 2006). Finally, BON cells and human EC cells express functional A_2B_ receptors that are positively linked to mechanotransduction pathways, described further later (Chin et al., 2012). Overall, BON cells, and various other EC cell lines have significant limitations as EC cell models, and there is no replacement for normal EC cells. However, and in particular BON cells, have been instrumental in delineating some of the fundamental properties of mechanosensation in EC cells as will be described further in this review.

### Mechanosensitivity

5-HT release from EC cells activates secretory and peristaltic reflexes that are necessary for lubrication, mixing movements during digestion, and propulsion of intestinal luminal contents. In general, the process of mechanotransduction has been the subject of intense investigation. The mechanisms operating in other cells (*endothelial, epithelial, dendritic cells, Merkel cells, glial cells, sensory neurons*) (Kunze et al., 2000; Weinbaum et al., 2011; Zagorodnyuk and Spencer, 2011) to convert the MS into an intracellular biochemical signal may be relevant to EC cells. Mechanotransduction in cells of intact tissues are regulated by their microenvironment that includes neighboring cells (i.e. epithelial cells, other enteroendocrine cells and immune cells in the case of EC cells), and the extracellular matrix (ECM). The connections between the cell-ECM are dynamically regulated by integrins (Teräväinen et al., 2013). Mechanotransduction is known to involve mechanosensitive ion channels (Arnadóttir and Chalfie, 2010). Mechanotransduction could be the result of a coordinated response between a variety of membrane molecules and microdomains, ion channels, receptors, G proteins, the cytoskeleton and lipid bilayer membrane supporting cell structure, caveolae, and adhesion molecules; membrane fluidity is an important mechanism as well (Weinbaum et al., 2011; Teräväinen et al., 2013; Ranade et al., 2014; Woo et al., 2014). Cells may respond to MS by activation of various intracellular signaling pathways leading to modulation of gene or protein expression and release of “mechanosignaling” molecules to regulate cell-to-cell communication and cellular functions. ATP, nitric oxide, prostaglandins or adenosine are *important mechanosignaling* molecules for such communication (Weinbaum et al., 2011).

The BON cell model was used to study the mechanisms and signaling pathways involved in mechanosensitivity, including *shear stress and pressure*. In the original study Kim et al. (2001a), rotational shaking was used as a MS to evoke 5-HT release in the cells, in intact guinea-pig colon or intact human colon. This type of stimulus is associated with shear stress and pressure, and increasing rotation from 0-100 rpms elicits a graded increase in 5-HT release without affecting cell viability (Kim et al., 2001a). The maximum shear stress calculated from the equation *T*_*max*_ = *a*√ρη*(2*π*f)* ^3^ for 60 rpm is 2.6 dyn/cm^2^ (Christofi, 2008). Use of a special laminar flow chamber to study shear stress indicates that 2.0 dyn/cm^2^ shear stress is sufficient to evoke release of 5-HT in BON cells {calculated using the formula *T* = *6*μ*Q/bh* ^2^*(dyn/cm*^2^*)*}. A parallel flow microchamber allows exposure of cells to fluid shear stress from 1 - 300 dynes / cm^2^ in a flow channel (2 mm wide) that is optically accessible through a coverslip-based window to monitor Ca^2+^ responses (Vilardaga et al., 2003; Hoffmann et al., 2005). This is worth pursuing in future studies to investigate impact of fluid shear stress on normal EC cell function.

With rotational shaking, the maximum hydrostatic pressure imposed on the cell surface by movement of assay buffer at 100 rpm is 1.33 mm Hg according to the Unit conversion Factor (1g/cm^2^ = 0.74 mm Hg; Kim et al., 2001a; Christofi, 2008). In fact, very high pressures in the noxious range are needed to stimulate 5-HT release from BON cells (e.g., 50–75 mm Hg) using a special chamber to increase pressure. Such high intraluminal pressures can occur in pathologic states of over distended gut or patients with severe constipation. In pathophysiological conditions, abnormal distension associated with painful sensation involves release of ATP to activate EC/enteric nervous system/motor pathways and visceral afferent pain pathways (Wynn et al., 2004; Burnstock, 2009).

Overall, BON cells are much more sensitive to “shear stress” (i.e., *produced by increasing fluid flow in tubular structures like the intestine*) than increase in “pressure” for releasing 5-HT. This information could only be obtained in isolated cells, since *in vivo*, multiple mechanical forces are operating at the same time in the gut during peristalsis, and it is not possible to separate the effects of *pressure, shear stress, distension, compression, deformation, or centrifugal forces* during peristalsis.

### P2Y_1_/G_α_q/PLC/IP_3_/IP_3_R-SERCA Ca^2+^ signaling pathway in mechanotransduction

MS released 5-HT from BON cells, as well as guinea pig or human jejunum EC cells during neural blockade with tetrodotoxin (Kim et al., 2001a; Linan-Rico et al., 2013a). In BON cells, it was shown that blockade of G_α_q and other G-proteins by GDP-β-S or use of a synthetic peptide derived from the COOH terminal tail of G_α_q which interferes with receptor—G protein coupling (with amino acid sequence, VFAAVKDTILQLNLKEYNLV) abolished mechanically evoked 5-HT release, whereas the NH_2_-terminal peptide (EEAKEARRINDEIERQL) had no effect. An antisense phosphorothioated oligonucleotide targeting G_α_q reduced G_α_q protein levels and abolished mechanically evoked 5-HT release without affecting expression of another G-protein, Gα_11_. These pharmacological observations suggest that receptor-G protein coupling is necessary for 5-HT release (Kim et al., 2001a) but do not completely rule out direct activation of G_α_q or PLC by physical forces. The study also pointed to a G-protein coupled P2Y receptor since MS releases both ATP and UTP that activate these receptors. MS of the phospholipid bilayer could potentially activate G proteins directly without the necessity of an agonist-occupied receptor (Gudi et al., 1998). It should be noted however, that it is difficult to identify the most upstream event in mechanotransduction, and which is the sensor or down-stream target without electrophysiological studies to identify the precise timing/kinetics of responses, i.e., activation of a mechanogated channel as described later. As suggested previously, *it is also* possible that different stimuli are detected by different mechanosensory—mechanotransduction pathways (Cooke et al., 2003) and this remains to be evaluated.

### Caveolin-associated cholesterol rich membrane domains in mechanotransduction

The cell membrane is a phospholipid bilayer interspersed with lipid rafts rich in cholesterol and sphingolipids. Caveolae are a subfamily of lipid rafts (Shaul and Anderson, 1998; Smart et al., 1999; Oh and Schnitzer, 2001; Chini and Parenti, 2004) that are likely involved in EC cell mechanotransduction—these structures are small (50–100 nm) flask-shaped plasma membrane invaginations rich in caveolins, sphingolipids, and cholesterol. They have been hypothesized to play a critical role in signal transduction by forming a scaffold on which ion channels, receptors, and signaling factors are anchored or assembled (Shaul and Anderson, 1998). There is convincing evidence that caveolae are implicated in shear stress mechanotransduction in vascular endothelial cells (Ando and Yamamoto, 2013).

In the BON cell model, mechanical stimulation elevates intracellular free Ca^2+^ levels and stimulates 5-HT release (that is a Ca^2+^-dependent process; Kim et al., 2001a; Cooke et al., 2003 review; Christofi et al., 2004b; Linan-Rico et al., 2013a). Indeed manipulations that disrupt caveolae inhibit both intracellular Ca^2+^ signals and the Ca^2+^ dependent 5-HT release in response to mechanical stimulation. Disassembly of caveolae with a cholesterol binding agent filipin or treatment with methyl-β-cyclodextrin (β-CD) to disrupt the caveolar structure by depleting membrane cholesterol suppresses both touch-induced Ca^2+^ responses and rotational shaking—induced 5-HT release. These agents also block the release of ATP in response to MS. Therefore disruption of caveolae is a critical determinant of mechanosensitivity, ATP and 5-HT release (Kim et al., 2007).

A novel ATP imaging technique has been used to demonstrate that highly concentrated ATP release (>10 μM ATP) occurs locally from caveolae-rich regions of the plasma membrane (caveolin-1, marker protein of caveolae); visualization of ATP release was done using biotin-luciferase protein attached to a biotinylated cell surface with streptavidin (i.e., *ATP release triggers the luciferin-luciferase chemiluminescence reaction*; Ando and Yamamoto, 2013). EC cells release ATP in response to mechanical stimulation (Linan-Rico et al., 2013a) and this mechanism deserves further consideration.

In endothelial cells, caveolin-1 is a scaffolding protein that holds G_α_q subunits in the inactive GDP-bound state until activation of G_α_q terminates association with caveolin-1 and releases G_α_q. As such one of the functions of caveolin-1-associated domains is presumed to be to concentrate and stabilize G_α_q (Li et al., 1995; Okamoto et al., 1998; Oh and Schnitzer, 2001; Chini and Parenti, 2004). In fact, activation of G_α_q is essential for mechanosensitive release of 5-HT in BON cells, because as noted earlier GDP-β-S or the antisense oligonucleotide to a specific sequence of G_α_q blocked mechanically-induced 5-HT release (Kim et al., 2001a; Cooke et al., 2003 review). This has not yet been evaluated in normal mouse or human EC cells. Methyl-β-cyclodextrin and filipin disrupt the structure of caveolae and the caveolin-1/G_α_q protein interactions (Liu et al., 1997; Hailstones et al., 1998). In the only study done, MS of BON cells uncoupled G_α_q from the caveolin-1 fraction as shown in co-precipitation studies and increased 5-HT release. Caveolin-1 transcripts and protein expression were detected in BON cells. Disassembly of caveolin-associated membrane domains using filipin or by cholesterol depletion with β-CD abolished 5-HT release. Therefore, caveolin-1 and caveolin-1 associated cholesterol rich membrane microdomains in the lipid bilayer are critical regulators in the mechanically evoked 5-HT release (Kim et al., 2007). This is consistent with other reports that altering membrane cholesterol levels alters the activity of G-protein coupled receptors (Gimpl et al., 1995, 1997, 2000).

### Down-stream 5′-nucleotide autocrine modulation of mechanosensitivity in EC cells

The P2Y_1_receptor is a critical component of the mechanotransduction pathway in sensory neurons and BON cells. P2Y_1_ purinergic receptors in sensory neurons contribute to touch-induced impulse generation (Nakamura and Strittmatter, 1996). In that study, a single cRNA derived from sensory neurons encoding a P2Y_1_ receptor renders *Xenopus laevis* oocytes mechanosensitive. Hence, in oocytes, the P2Y_1_ is required for mechanotransduction. The P2Y_1_ mRNA is expressed in large-fiber DRG neurons (unlike P2X_3_, in small-fiber sensory neurons). Therefore, a transduction system based on P2Y_1_ that may form the sole basis for generating nerve impulses might be an essential early step in touch-sensitivity. Studies in BON cells identified a P2Y_1_/G_α_q/PLC-IP_3_-Ca^2+^ signaling pathway as an essential transduction pathway involved in converting physical forces generated by mechanical stimulation to a physiological response 5-HT release. This pathway likely represents an essential component of the mechanotransducer assembled in the plasma membranes of BON cells tethered with other components. Again, the significance of the P2Y_1_ pathway needs to be confirmed in normal EC cells.

### Are GPCRs mechanosensors?

G protein coupled receptors (GPCRs) have a myriad of roles in transducing extracellular signals into cellular responses. Membrane potential may regulate GPCRs. A voltage-induced conformational change in the receptor may alter (enhance) its ability to couple the G protein and influences its affinity for an agonist (Mahaut-Smith et al., 2008). For a majority of Gq-coupled receptors (e.g., P2Y_1_), depolarization enhances downstream Ca^2+^ mobilization. EC cells have been shown to be excitable cells displaying Na^+^ sensitive action potentials (Strege et al., 2016) and therefore such an interaction deserves further consideration. The P2Y_1_—membrane potential synergistic interaction might be important at low levels of agonist where depolarization can still evoke substantial Ca^2+^ mobilization (Voltage-dependence of GPCRs or their signaling pathways).

Mechanical stress can activate GPCRs (i.e., angiotensin II type 1 receptor) independently of agonist being present. That is, GPCRs can potentially serve as mechanosensors. First, stretching the cell membrane may directly change the conformation of the receptor to an active state. Second, mechanical stretch could activate specific mechanical sensors, which then activate the receptor from insight the cell, i.e. potential stretch sensors are integrins, stretch-sensitive ion channels (Sachs, 2010; Delmas et al., 2011; Reed et al., 2014). G protein coupled receptors in endothelial cells sense fluid shear stress (Chachisvilis et al., 2006). It was shown that changes in cell membrane tension and membrane fluidity affect conformational dynamics of GPCRs, suggesting that GPCRs are involved in mediating primary mechanochemical signal transduction in endothelial cells. They used time-resolved fluorescence microscopy and GPCR conformation-sensitive FRET (Fluorescence Resonance Energy Transfer), and were able to show that stimulation of cells with fluid shear stress, hypotonic stress, or membrane fluidizing agent leads to an increase in activity of bradykinin B2 GPCR in the cells. Such studies are needed in EC cells, and on the effects of changes in lipid bilayer environment on GPCR conformational dynamics. GPCR are a major target of drug development, and a detailed analysis of mechanochemical signaling via GPCR pathways may be relevant for development of new GI medications targeting 5-HT abnormalities in IBD or IBS.

Fine-tuning of GPCR activity occurs via receptor-interacting proteins (Ritter and Hall, 2009). The angiotensin II type 1 (AT1) receptor was the first GPCR implicated to be mechanosensitive (Mederos y Schnitzler et al., 2011). Indeed, MS (shear stress) can activate G proteins reconstituted into liposomes (in the absence of any other potential mechanosensors) suggesting that the lipid bilayer membrane plays a key role in mediating mechanomechanical signal transduction (Gudi et al., 1998). By using time-resolved fluorescence microscopy and GPCR conformation-sensitive FRET, it was shown that changes in cell membrane tension due to fluid shear stress and membrane fluidity could cause ligand-independent conformational transitions of a GPCR to an active conformation in endothelial cells. It has been suggested that GPCRs could mediate primary mechanochemical signal transduction (Chachisvilis et al., 2006).

There is a significant sub-population of EC cells that do not require endogenous ATP or other nucleotides for mechanotransduction as demonstrated by indirect pharmacological experiments (Linan-Rico et al., 2013a). Therefore, MS (touch/stretch) is blocked in 77% of BON cells by a 5′ectonucleotidase apyrase, is enhanced in 63% of cells by ARL67156, is blocked in ~50% of cells by a P2Y_1_ antagonist MRS2179 or a general P2 antagonist PPADS. Whether MS can directly activate the P2Y_1_ GPCR (or other GPCRs, i.e., A_2A_/A_2B_; Christofi et al., 2004b) or whether activation of mechanogated channels such as Piezo 2 (see later) can activate 5-HT release directly without ATP release in EC cells remains unknown. It is possible to use FRET technology to study G-protein—receptor interactions in response to mechanical stimulation.

Mechanical stress can operate to increase membrane fluidity and affect mechanotransduction. This is likely involved in EC cell mechanotransduction, but there is as yet no evidence to support it. Measurements of membrane fluidity with the fluorescent dye 4-(dicyanovinyl)julolidine have provided direct proof that mechanical stress increases membrane fluidity in an intensity-dependent manner in human blood vessel endothelial cells (Haidekker et al., 2000; Butler et al., 2001).

Findings in BON cells established that endogenous purines are critical determinants of 5-HT release evoked by MS. However, as described later, mechanotransduction is a complex process that also requires the activation of mechanogated channels. A proposed model of purinergic mechanosensory signaling in EC cells is illustrated in Figure 1. MS releases ATP (and UTP) to act in an autocrine manner to modulate 5-HT release. It has been proposed that purines provide a mechanism of fine-tune modulation of 5-HT release in regulating gut mechanical reflexes. ATP could also act directly *in vitro* to activate reflexes in rodents (Christofi et al., 2004a; Cooke et al., 2004). MS releases ATP (or ADP) to activate a slow P2Y_1_-Gq/PLC/IP3-IP3R—SERCA pump—Ca^2+^ dependent pathway to evoke release of 5-HT. In a separate population of human EC cells, ongoing activation of a slow P2Y_12_-G_*i*/*o*_AC/cAMP/PKA signaling pathway by ADP (or ATP) inhibits 5-HT release that is expected to attenuate reflexes. In addition to these GPCRs (P2Y_1_ and P2Y_12_), ATP can activate a fast ATP-gated P2X_3_ (or P2X_1_) channel to stimulate 5-HT release (Linan-Rico et al., 2013a).

A study by Linan-Rico et al. (2014) showed that several ionic conductances are implicated in the regulation of 5-HT release in BON cells. Patch-clamp and Ca^2+^ studies provided evidence for regulation of 5-HT release by UTP. Data suggest that UTP activates a predominant P2Y_4_ receptor to elicit Ca^2+^ responses by activating a Gq/PLC/IP_3_/IP_3_R/SERCA Ca^2+^ pump—signaling pathway to stimulate 5-HT release. UTP stimulated voltage-sensitive Ca^2+^ currents, (I_ca_), caused V_m_-depolarization and inhibited I_K_ (not I_A_) currents. Membrane depolarization evoked by UTP was linked to a PLC/I_Kv_ pathway, but it was not linked to changes in intracellular Ca^2+^ levels in BON cells (Linan-Rico et al., 2013b, 2014). UTP stimulated 5-HT release in BON or freshly isolated human EC cells (identified by their expression of tryptophan hydroxylase 1 (Tph1), 5-HT, and mRNA for P2Y_4_/P2Y_6_) (Linan-Rico et al., 2013b, 2014). Overall, UTP-gated signaling pathways are considered to be important regulators of 5-HT signaling. The role of UTP in mechanosensory signaling in EC cells is largely unexplored.

Release of ATP or UTP from surrounding epithelial cells contributes to paracrine stimulation of EC cells (Figure 1). Cell to cell communication in response to mechanical stress occurs via bilateral release of ATP and UTP in polarized epithelia (Homolya et al., 2000). ATP release (Linan-Rico et al., 2013a) and adenosine release (Christofi et al., 2004b) is shown to occur during MS of BON cells, but although it is possible that UTP is also released by EC cells and surrounding epithelial cells, it has not yet been shown to occur. Moreover, it is not known whether the same or different mechanosensory—mechanotransduction pathways, including those involved in releasing purines (ATP and UTP) operate in EC cells and surrounding epithelial cells during peristalsis. Furthermore, it is also not clear what physiological conditions favor autocrine (EC release of purines) or paracrine (epithelial release of purines) purinergic regulation. Our hypothesis is that autocrine regulation provides local, moment-by-moment fine tune modulation of 5-HT release, whereas paracrine regulation by surrounding epithelia provides more global regulation of 5-HT secretion to coordinate the movements of large portions of the gut.

### Adenosinergic modulation of mechanically evoked 5-HT release

The tonic ongoing release or mechanically evoked release of endogenous adenosine is a critical determinant of differential activation of adenosine receptors with potentially important implications for gut mechanosensory reflexes (Christofi et al., 2004b). Adenosine A_1_, A_2*a*_, A_2*b*,_ and A_3_ receptors are expressed in BON cells as revealed by pharmacological, RT-PCR, immunochemical and western blot techniques. Adenosine release to MS was detectable by the LC-MS/MS technique. MS evoked adenosine release, and the nucleoside (adenosine) uptake inhibitor {S - (p-nitrobenzyl)-6-thio-inosine, NBTI} enhanced mechanically evoked adenosine release. Treatment with an adenosine / nucleoside uptake inhibitor NBTI enhances adenosine release to ~100 nM (from 30 nM) in the medium bathing the cells and suppressed mechanically evoked 5-HT release. The enzyme adenosine deaminase that catalyzes the breakdown of adenosine caused a concentration-dependent increase in 5-HT release, suggesting that endogenous adenosine is providing an ongoing inhibitory modulation of 5-HT release. The resting level of endogenous adenosine acts as a “physiological break” on baseline 5-HT release by activation of A_3_ receptors. These and other observations support the hypothesis that adenosine is a key autocrine modulator of 5-HT release. Endogenous adenosine provides dual modulation of mechanically evoked 5-HT release via Ca^2+^ dependent inhibitory A_3_ and A_1_ linked signaling pathways, or cAMP-dependent- excitatory A_2*a*_ or A_2*b*_ receptor linked pathways. In the physiological setting, mucosal stroking, stretch or other mechanical stimuli release adenosine, ATP (or UTP?) to modulate mechanosensitive secretory reflexes (Cooke et al., 1999, 2004; Christofi et al., 2004a). Adenosinergic signaling represents a key regulatory mechanism linked to 5-HT release from EC cells, and they have important implications for chemo- or mechanosensory reflexes in the gut and possibly for tumor therapy (i.e., *since they were also expressed in human gastric and intestinal 5-HT containing carcinoid tumors*).

### Effects of purines on mechanically-evoked mucosal secretory reflexes

5-HT release stimulates the enteric nervous system (ENS) to influence neural secretory reflexes, peristaltic activity, and plays a role in digestive motility (i.e., segmenting pattern of motility involved in mixing movements during digestion), ascending contraction and descending relaxation reflexes (reviewed by Mawe and Hoffman, 2013). Recent studies raised doubt as to whether EC cell 5-HT is essential for colonic peristalsis or colonic migrating motor complexes (CMMCs). In this regard, colonic peristalsis was shown to occur without endogenous 5-HT. Therefore, it was shown that intestinal transit and colonic bead expulsion was not changed in Tph1 knockout (KO) mice that cannot synthesize or release 5-HT from EC cells. TPh1 KO mice can still synthesize 5-HT via Tph2 in myenteric (and raphe) neurons. Tph1 can be found in EC cells, but also in some immune cells and other cell types in the periphery (Amireault et al., 2013). Specifically, these mice did not have any inhibitory deficits in intestinal transit (Yadav et al., 2010; Li et al., 2011) and CMMCs still occurred in the Tph1 KO mice (Heredia et al., 2013). Along these lines, Spencer and co-workers were still able to trigger peristalsis by stretching the tissue after removing the mucosa, and still record spontaneous and evoked CMMCs (Keating and Spencer, 2010; Spencer et al., 2011). In mucosa-free preparations, release of 5-HT was abolished.

In the rat distal colon mechanically evoked reflex electrogenic chloride secretion is triggered by endogenous nucleotides (ATP, UTP, and UDP) acting at P2Y_1_, P2Y_2_, and P2Y_4_ receptors (Christofi et al., 2004a). These receptors are expressed on both EC cells and submucosal neurons, and therefore, both autocrine activation of EC cells to secrete 5-HT, as well as direct activation of neural P2Y receptors are suggested to be involved in secretomotor functions. Species differences exist in purinergic modulation of neural reflex chloride secretion, and in the guinea-pig, MS by stroking the mucosa releases nucleotides that act predominantly at neural P2Y_1_ receptors to trigger chloride secretion (Cooke et al., 2004). In the rat, other purinergic receptors are also involved in regulating secretomotor function (Christofi et al., 2004a). Overall, release of ATP or other nucleotides by MS can trigger secretomotor reflexes in rodents. Similar studies in human intestine are lacking.

### Mechanogated Piezo2 channels

The identity of the mechanosensor(s) in EC cells remains unclear. Mechanosensitivity may be conferred by the open or closed state of a mechanogated ion channel, a receptor such as the P2Y_1_ receptor, G-proteins, caveolae, integrins, or receptor tyrosine kinases. Recent evidence suggests Piezo 2 mechanogated channels are an important component of mechanosensitivity in EC cells.

The EC cell is an important mechanosensory cell in the GI epithelium, and it is well established that mechanical stimulation causes 5-HT release. Contractile activity of the gut and mucosal compression of the gut wall triggers release of mucosal 5-HT (Bertrand, 2006). In human gut *in vitro* preparations, mechanical stimulation to mimic mechanical forces during peristalsis induces 5-HT release (Linan-Rico et al., 2013a). However, mechanical stimulation, mucosal compression or mechanical deformation can distort or stimulate several cell types in the gut wall besides EC cells, including epithelial cells (Burnstock, 2008a,b, 2009), other enteroendocrine cells, mechanosensitive AH neurons of the myenteric plexus (Kunze et al., 2000) and enteric glial cells (Zhang et al., 2003; Liñán-Rico et al., 2016). Direct evidence for mechanosensitivity in EC cells was obtained from BON cells for Ca^2+^responses and 5-HT release (Kim et al., 2001a; Linan-Rico et al., 2013a), human EC cells isolated from surgical specimens for 5-HT release (Chin et al., 2012) and the QGP-1 EC cell model for mechanosensitive currents (Wang et al., 2016).

While the EC cell mechanotransduction mechanisms have received their due attention, the primary mechanosensor has been an important unanswered question. Primary mechanotransducers in other systems (e.g., hearing, touch) rely on mechanosensitive ion channels, which couple mechanical forces to changes in ion conductances (Arnadóttir and Chalfie, 2010). The EC cell has substantial developmental (Yan et al., 2001; Li H. J. et al., 2011; Roach et al., 2013; Wright et al., 2015) and functional similarities (Raybould et al., 2004; Nakatani et al., 2014; Chang et al., 2016) to the Merkel cells, which are light touch sensors in the skin. During development for example, there is a requirement for Atoh1 for secretory cell lineage commitment in mouse intestine and Merkel cells. Loss of *Atoh1* in progenitor cells prevents differentiation of secretory cells, including enteroendocrine cells and EC cells of the gut and Merkel cells. Recent discoveries have unveiled the mechanosensitive ion channel Piezo2 as critical primary mechanosensor for Merkel cell mechanosensitivity (Ikeda and Gu, 2014; Ranade et al., 2014; Woo et al., 2014). These studies prompted the investigation of Piezo2 channel involvement in EC cell mechanotransduction. Piezo2 was found specifically expressed in EC cells of human and mouse small bowel (Wang et al., 2016). An EC cell model of pancreatic origin, QGP-1had mechanosensitive currents with biophysical and pharmacologic properties of Piezo2 channels and these currents were nearly eliminated by Piezo2 siRNA. Importantly, stretch-induced 5-HT release was inhibited by the pharmacologic and siRNA inhibition of Piezo2 (Wang et al., 2016). In mouse small bowel, pharmacologic blockade of Piezo channels resulted in a substantial decrease of 5-HT mediated pressure-induced secretion. Therefore, Piezo2 is an important EC cell mechanosensitive ion channel. However, many questions remain.

*Firstly*, does Piezo2 serve as the primary mechanosensitive ion channel, or does it work in concert with other mechanosensors? Previous studies have identified several membrane proteins that may collaborate with Piezo2. Transient receptor potential channel TRPA1 is another cation selective ion channel, which is specifically expressed in EC cells (Nozawa et al., 2009). TRPA1 is known to be critical for mechanosensation in other cellular systems (Corey et al., 2004). Interestingly, the light touch sensory neurons (Aβ) use the Piezo2 as primary mechanosensors (Ranade et al., 2014). TRPA1 alters mechanosensory adaptation to long-lasting stimuli (Kwan et al., 2009), suggesting that TRPA1 collaborates with Piezo2. However, the involvement of TRPA1 channel in the molecular mechanism of mechanosensation remains unknown. Similarly, P2X are purine gated cation ion channels involved in EC cell mechanosensation (Christofi, 2008; Linan-Rico et al., 2013a). While P2X channels are not themselves mechanosensitive, it is well known that ATP release is a common response to mechanical stimulation in many mechanosensory systems. In the bladder epithelium, Piezo activation is critical for ATP release, and therefore it may provide a positive feedback loop upon mechanical stimulation (Miyamoto et al., 2014). Caveolae are well known to be involved in mechanosensation (Yu et al., 2006), and specifically in the BON cells caveolae disruption alters mechanosensory response (Kim et al., 2007). Mechanosensitive ion channels, such as Piezo, have been previously shown to localize within microdomains (Gottlieb et al., 2012), where their mechanosensitivity properties are strongly affected. In addition to the Piezo2 channels, Piezo1 mechanosensitive ion channels have recently been shown to be important for the response to static forces by GI epithelial cells (Eisenhoffer et al., 2012). In chondrocytes, Piezo1 and Piezo2 channels cooperate to produce a mechanosensitive response (Lee et al., 2014). However, it is currently not known whether Piezo1 channels are expressed in EC cells and if expressed, if they cooperate with Piezo2 channels. In all, Piezo2 has emerged as an important EC cell mechanosensor, but it is not yet clear whether it works within a mechanosensitive complex.

*Secondly*, what is the downstream mechanism of mechanical Piezo2 activation in EC cells (Figure 1)? Piezo2 is a non-selective cation ion channel with rapid activation and inactivation kinetics (Coste et al., 2010; Ranade et al., 2014). Upon mechanical stimulation Piezo2 opens and inactivates within roughly 10 ms, which is significantly shorter than the subsequent 5-HT release (Bertrand, 2004). This implies that while Piezo2 may directly stimulate 5-HT secretion due to Ca^2+^ influx through Piezo2 leading to exocytosis, signal amplification is required for full response (Figure 1). This may come via either or both, ionotropic or metabotropic pathways, both of which exist in EC cells. As described above, it is now established that Piezo channels are upstream of ATP release by regulating the density of Ca^2+^ influx, but the details of this mechanism remain unknown (Miyamoto et al., 2014; Cinar et al., 2015). In the Merkel cells, Piezo2 activation leads to a complex downstream response, which culminates in activation of voltage-gated calcium channels (Nakatani et al., 2014). In EC cells, several possibilities exist. First, Piezo2 activation leads to the initial depolarization that activates voltage-gated ion channels, such as Na_*V*_ or Ca_*V*_ channels, which provide the final common pathway to Ca^2+^ influx that is required for exocytosis. Second, Piezo2 may conduct enough calcium to initiate a calcium-dependent-calcium release from the internal stores. For example, intracellular calcium is known to be important for the mechanosensitive response in the human EC cell model BON (Kim et al., 2001a).

In summary, Piezo2 mechanosensitive ion channels are critical for 5-HT release but several important details on the EC cell primary mechanosensory complex and molecular signaling mechanisms downstream of the Piezo2 channels remain unknown. Figure 1 shows how Piezo 2 channels could be involved in mechanotransduction leading to 5-HT release.

### Impact of inflammation on purinergic modulation of 5-HT release

The topic of inflammation and alterations in 5-HT availability has been previously reviewed (Coates et al., 2004; Gershon, 2004; Manocha and Khan, 2012; Mawe and Hoffman, 2013). Briefly, 5-HT availability is increased in human inflammatory diseases and in animal models of gut inflammation (Coates et al., 2004; Manocha et al., 2012; Mawe and Hoffman, 2013). Emerging evidence suggests abnormal regulation of 5-HT in GI disorders and IBD (Manocha et al., 2012). Studies of EC cell numbers, tryptophan hydroxylase 1 (Tph1) mRNA levels, serotonin transporter (SERT) expression and/or 5-HT synthesis indicate alterations in 5-HT signaling and bioavailability in IBS and IBD (Coates et al., 2004; Manocha et al., 2012). Such effects are observed in ulcerative colitis (UC), Crohn's Disease (CD), diarrhea-predominant IBS (IBS-D) and Constipation-predominant IBS (IBS-C). Inflammation of the intestinal mucosa alters 5-HT signaling in both humans and animal models (Magro et al., 2002; Wheatcroft et al., 2005; Ghia et al., 2009). In IBS, several studies reported associations of symptoms of IBS and numbers of EC cells, expression of mRNA levels of Tph1, and SERT expression in mucosal biopsy; 5-HT content is also altered in IBS (Miwa et al., 2001; Coates et al., 2004; Spiller, 2008; Camilleri, 2009; Malinen et al., 2010; Cremon et al., 2011). Alterations in 5-HT signaling were also shown to be associated with colorectal cancer, diverticular disease (Costedio et al., 2008) and celiac disease (Wheeler and Challacombe, 1984; Coleman et al., 2006) as well. In some cases, excess release of 5-HT from EC cells such as occurs with the chemotherapeutic agent cisplatin can cause intense GI discomfort and vagal afferent stimulation of 5-HT_3_ receptors can lead to nausea and vomiting (Tyers and Freeman, 1992; Gale, 1995; Hillsley and Grundy, 1999).

Localization of 5-HT expressing EC cells in the intestinal mucosa provides direct access of oral medications to modulate 5-HT release or synthesis. Clinical trials with a small molecule inhibitor of Tph1 to suppress synthesis of 5-HT in EC cells was shown to be efficacious in alleviating the symptoms of diarrhea-predominant IBS—it increased stool consistency, relieved pain and discomfort in the patients and reduced bioavailability of 5-HT (Sanger, 2008; Brown et al., 2011). Experimental medicines given by oral administration show efficacy in pre-clinical animal models of IBD and IBS. A better understanding of purinergic regulation of 5-HT release in health and disease may also provide potential novel therapeutic targets on EC cells. In fact, oral drugs targeting adenosine receptors are being pursued in clinical trials for rheumatoid arthritis, CD, bladder pain syndrome, uveitis, IBS and functional dyspepsia (Ochoa-Cortes et al., 2014).

Inflammatory mediators from immune cells such as IL-13 regulate EC cell hyperplasia and 5-HT production in the gut (Manocha et al., 2013). In IL-13^−/−^ deficient mice infected with *Trichuris muris* parasite, the numbers of EC cells and 5-HT amount were lower compared to wild-type mice after infection. IL-13^−/−^ mice fail to clear the parasite from the gut unlike wild-type mice. Treatment of control or IL-13^−/−^ mice with IL-13 had the opposite effect to increase EC cell numbers and 5-HT amount. BON cells produced more 5-HT in response to IL-13 (Manocha et al., 2013). This provides proof of concept for immunologic control of 5-HT release in the gut that may be relevant in GI disorders, IBD or GI infections and may provide a paradigm for investigation of the impact of immune/inflammatory mediators on mechanotransduction and 5-HT release from EC cells.

Available data suggest that vagal afferent signaling via 5-HT receptors is involved in regulation of satiety and hunger in humans. Nutrients (i.e., glucose, carbohydrate breakdown products, lipids/free fatty acids) can activate a vago-vagal reflex to inhibit gastric emptying (Raybould et al., 2003) and also contribute to satiety signals to suppress food intake by simultaneous activation of 5-HT_3_ and CCK_1_ receptors. In the inflamed intestine 5-HT can activate extrinsic spinal afferents to transmit pain signals to the brain (Mawe and Hoffman, 2013).

5-HT_3_ receptors on intrinsic or extrinsic nerve fibers of the mucosa are activated by 5-HT release from EC cells (Grundy, 2008; Mawe and Hoffman, 2013). Intrinsic primary afferent AH neurons of the myenteric plexus (i.e., IPANs) projecting to the mucosa can be activated by 5-HT application to the mucosa to activate 5-HT_3_ receptors on the afferent process (Bertrand et al., 2000). 5-HT_3_ antagonists are effective drugs for IBS-D to treat the diarrhea (Houghton et al., 2000; Camilleri et al., 2001; review by Fayyaz and Lackner, 2008). Inhibition of motility by blocking excitatory 5-HT_3_ receptors on intrinsic afferents and other neurons in the ENS contributes to the anti-diarrheal effect. 5-HT_3_ antagonists are also effective against GI discomfort and pain sensation by blocking 5-HT_3_ receptors on both intrinsic and extrinsic afferent fibers. 5-HT_3_ agonists are also being tested for the treatment of constipation for IBS-C for their ability to promote motility (Choung et al., 2008).

### Impact of pathologic conditions, GI diseases and GI disorders on gut mechanotransduction

*In vivo* serotonin release has been induced by intestinal smooth muscle contraction and other mechanical forces (Kirchgessner et al., 1996; Bertrand, 2006). EC cells, epithelial cells and other cells of the intestinal tract (*enteric glial cells, ICCs, mechanosensitive neurons*) experience a “myriad of physical forces” during peristaltic activity including mucosal pressure, deformation, compression, shear stress, strain, centrifugal forces (i.e., *during segmenting movements of the digestive phase*) and other forces. GI disease, disorders and pathologic conditions alter these forces leading to adverse effects in the biology of EC (or other cells). For example, during chronic intestinal inflammation, an increase in intraluminal pressures can alter gut physiology (Kellow and Phillips, 1987; Brodribb et al., 1997; Basson et al., 2000). Abnormalities in these forces and hence “mechanotransduction” in EC cells can result in pathophysiological changes and even contribute to motility disorders (Kuemmerle, 2000). Inflammation (and injury) from an inflammatory bowel disease (i.e., Crohn's Disease) or bowel wall edema caused by surgical procedures can further elevate intraluminal pressures and cause abnormal motility (Granger and Barrowman, 1983). Following intestinal surgery, the bowel takes up fluid and becomes edematous, and intra-abdominal pressure can increase as high as 15–40 mm Hg above normal for several days (Williams and Simms, 1997; Madl and Druml, 2003). Furthermore, in IBD, substantial increase in colonic blood flow (i.e., 2 to 6-fold) can increase capillary pressures (Hultén et al., 1977) and also interstitial pressures. In IBS patients, intraluminal pressures in the small bowel can reach 50 mm Hg (Kellow and Phillips, 1987). Mucosal atrophy is a common feature in IBD (Surawicz et al., 1994), in states of ileus or after prolonged fasting. Normal gut forces are altered with mucosal atrophy, and aberrant peristaltic contractions could be a contributing factor in GI pathology. Furthermore, during open intestinal surgery, physical manipulations of the bowel activate mechanosensitive cells of the gut wall. Mechanical forces in the gut can also influence epithelial cancers (Basson et al., 2000; Fernandez-Sanchez et al., 2015). Therefore, pathologic conditions of the GI tract influence mechanotransduction, and hence ATP and 5-HT release from EC cells with important consequences to serotonergic signaling in the gut.

### Role of 5-HT in the pathogenesis of intestinal inflammation

EC derived 5-HT plays a key role in the pathogenesis of intestinal inflammation and activation of dendritic cells in the mucosa has been suggested as a mechanism (Li et al., 2011). First, in DSS or DNBS colitis, reduction in the availability of mucosal 5-HT by deletion of Tph1, the enzyme that synthesizes 5-HT reduces the severity of inflammation (Ghia et al., 2009). Similarly, colitis animals treated with a selective Tph1 inhibitor LP533401 to suppress 5-HT synthesis had reduced intestinal inflammation Margolis et al. (2011). The severity of colitis is increased if the animals are treated with the 5-HT precursor 5-hydroxytryptophan to bypass Tph1 in the Tph1 KO mice. In SERT deficient mice, higher bioavailability of 5-HT aggravates the intestinal inflammatory response in animal models of colitis, i.e., TNBS or IL10 KO mice (Bischoff et al., 2009; Haub et al., 2010).

5-HT release is tightly regulated by purinergic autocrine and paracrine mechanisms and purinergic signaling is very sensitive to inflammation (Ochoa-Cortes et al., 2014). Emerging evidence indicates abnormal purinergic signaling in EC cells, i.e., A_2B_, P2X_3_, and 5-HT release although our knowledge remains very limited. ATP-gated - P2X_2/3_ and P2X_3_ channels represent a potential therapeutic target for analgesia/pain (Antonioli et al., 2008; Burnstock, 2008a,b; Ochoa-Cortes et al., 2014). ATP release from EC cells (or from surrounding epithelial cells) can activate these ATP-gated P2X channels on afferent fibers in the mucosa to transmit pain sensation to the brain (Burnstock, 2008a,b, 2009). Wynn et al. (2004) showed that the purinergic P2X component of mechanosensory transduction in response to *in vivo* luminal distension is increased in experimental colitis. P2X receptors are also linked to mechanosensitivity in BON cells and P2X_3_ receptors are expressed on human colonic EC cells identified by their 5-HT immunoreactivity and a P2X agonist α, β-MeATP can induce 5-HT release (Linan-Rico et al., 2013a). Multiple mechanisms have been proposed to regulate ATP release in a variety of cells, and among them, pannexin and connexin channels are receiving a great deal of attention (Huang et al., 2007; Anselmi et al., 2008; Beckel et al., 2014; Lohman and Isakson, 2014). The mechanisms regulating ATP release from EC cells are unknown in normal or inflamed states.

Discrete alterations in P2X_3_ and A_2B_ purine receptor expression occur in EC, epithelial cells and lamina propria from sigmoid colon of 11 UC surgical cases compared with 10 non-inflamed diverticulitis controls. A striking effect of mucosal inflammation in UC is the down-regulation of P2X_3_R. Detectable P2X_3_-immunoreactivity in hEC (5-HT^+^) cells was reduced from 15% to <0.2% of cells, whereas expression was not reduced in HuC/D^+^ neurons or inflammatory cells. In contrast, adenosine A_2B_-immunoreactivitity was reduced in both EC cells and neurons. The functional consequence of down-regulation of P2X_3_ (or A_2B_) receptors in UC is not known. In a colitis model, there is a loss of P2X-purinergic vascular regulation in mouse colon, but that was associated with up regulation of CD39, an enzyme that reduces the availability of ATP for activating P2X receptors (Neshat et al., 2009). P2X receptor mediated visceral hyperalgesia occurs in a rat model of visceral hypersensitivity (Xu et al., 2008). Functional upregulation of P2X_3_R rather than down regulation occurs in the chronically compressed dorsal root ganglion of the rat (Xiang et al., 2008). In our UC study described above, UC caused down regulation of A_2B_, and in 68% of EC cells A_2B_ was not detectable, where as in FACS-sorted human EC (hEC) cells isolated from surgical specimens in CD patients it was shown to cause upregulation of A_2B_ (Chin et al., 2012). Our study did not determine if in the remaining 32% of cells still expressing A_2B_ in UC specimens, it was up regulated. A_2B_ receptors are up regulated in both UC and CD biopsy, but the cell types were not identified (Rybaczyk et al., 2009). Differences in A_2B_ expression in EC from UC and CD could be due to differences between diseases (i.e. CD vs. UC), severity, chronicity or treatment of disease. Also, these cells are very sensitive to mechanical stimulation, and unavoidable EC cell handling, enzymatic dispersion, and mechanical agitation of EC cells from CD during isolation and FACS sorting (compared to intact EC cells in mucosa of UC) may affect their expression in different ways.

Despite the evidence for abnormal regulation of 5-HT in various diseases, it remains unknown how these changes in 5-HT occur (5-HT content, purinergic receptors, abnormal purinergic regulation associated with 5-HT release, content, Tph1 mRNA, etc.). Our hypothesis is that abnormal purinergic modulation of 5-HT release from EC cells is involved in the pathophysiology of several different GI disorders including IBS and IBD. Our knowledge of how inflammation leads to abnormal 5-HT signaling in the gut is marginal, and the role of purines, Piezo2 channels, GPCRs and other molecular components illustrated in Figure 1 deserves serious attention.

Emerging evidence from recent studies indicates that 5-HT contributes to the pathogenesis of intestinal inflammation. EC cell specific KO mice are proposed to be used in determining if changes in purinergic modulation of 5-HT release can influence the extent of gut inflammation. It may be a better/alternative strategy to figure out how to selectively reduce 5-HT release and bioavailability to limit the inflammatory response, rather than eliminating synthesis of 5-HT in EC cells that is important in so many physiological functions in and outside the gut. It is proposed that targeting purinergic modulation of 5-HT release and signaling at the level of the EC cell is a potential therapeutic strategy to treat GI Disorders and IBD, but this remains to be proven.

### Adenosinergic A_2B_ mechanosensitivity in IBD

A recent study by Chin et al. evaluated the role of mechanical forces and adenosine in the regulation of human intestinal EC cell 5-HT secretion in normal and Crohn's (IBD) EC cells (Chin et al., 2012). Human EC cells express stimulatory A_2B_ receptors and MS using a rhythmic flex model induces A_2B_ activation. An adenosine agonist NECA stimulated, whereas an A_2B_ receptor antagonist MRS1754 inhibited secretion of 5-HT, associated with corresponding changes in intracellular cAMP levels and pCREB. MS induced 5-HT secretion that could be inhibited by the A_2*B*_ antagonist and amplified by NECA. Normal and IBD-EC cells responded to A_2B_ receptor activation and A_2*B*_ receptor activation and A_2B_ antagonists could block mechanical evoked 5-HT secretion. This confirmed earlier findings on the role of A_2B_ receptors in mechanosensory signaling in the BON cell model (Christofi et al., 2004b). MS activated a PKA dependent 5-HT secretory pathway, and PKA/MAPK/IP_3_-dependent transcription. They also found that IBD-EC cells from Crohn's mucosa compared to normal mucosa from diverticulitis patients, and neoplastic EC cells (KRJ-1) overexpressed A_2B_ (and A_2A_) receptors and released more 5-HT (Chin et al., 2012).

Further studies can identify abnormal purinergic mechanosensory signal transduction mechanisms in EC regulating 5-HT release in UC and CD and in animal models of IBD or functional GI disorders. A bigger challenge is to develop more sophisticated approaches to evaluate impact of abnormal purinergic mechanosensory signaling in EC cells in intestinal reflexes (i.e. *mucosal secretory reflexes, digestive motility, ascending and descending reflexes*), as well as visceral pain sensation in IBD and IBS.

### Purinergic mechanosensory transduction and visceral pain and interactions with 5-HT

Visceral pain occurs in various diverse disorders including IBS, renal colic, dyspepsia, IBD, dysmenorrhea, interstitial cystitis, and angina. A subset of IBD patients in clinical remission without any signs of intestinal inflammation continue to experience pain and visceral hypersensitivity (IBS-like symptoms). Burnstock proposed the purinergic mechanosensory transduction hypothesis of visceral pain—the main concept is that purinergic mechanosensory transduction occurs in visceral tubes (ureter vagina, salivary and bile ducts and gut) and sacs (urinary bladder, gall bladder, lungs). Specifically in the gut, ATP released from epithelial cells during distension acts on P2X_3_ or P2X_2/3_ receptors to modulate peristalsis via intrinsic sensory reflexes (involving the ENS) or subepithelial sensory nerves transmitting pain signals via the dorsal root ganglia and spinal afferents to the central nervous system (CNS). Evidence for the hypothesis was obtained from a rat pelvic sensory nerve-colorectal preparation. By distending the colorectum, there was pressure-dependent increase in ATP release from epithelia lining the mucosa, and causes pelvic nerve excitation. ATP released during extreme (colic) distension acts on P2X_3_ and/or P2X_2/3_ receptors on high-threshold extrinsic sensory nerve fibers transmitting pain signals to the CNS (Burnstock, 2009). ATP release and P2X receptor mediated nociceptive sensory nerve responses are enhanced in a model of colitis (Wynn et al., 2004). P2X receptor mediated visceral hyperalgesia has also been reported in a rat model of chronic visceral hypersensitivity (Xu et al., 2008). Experimental evidence in the ferret esophagus suggests that ATP sensitization of vagal afferents to MS is also implicated in visceral hypersensitivity in non-erosive reflux disease (Page et al., 2002; Banerjee et al., 2006; Knowles and Aziz, 2008). ATP interacts with other mediators that can activate pelvic afferent fibers in the colorectum including 5-HT, bradykinin, prostaglandins and substance P (Barthó et al., 1999; Wynn and Burnstock, 2006). As shown in Figure 2, ATP release from epithelial cells or EC cells can have a paracrine (or autocrine) effect on 5-HT secretion and influence pain signals in an indirect way as well. 5-HT secretion acts on 5-HT_3_ receptors on extrinsic afferents transmitting pain information to the CNS. Furthermore, TRPV1 channels are sensitized by ATP released during distension, especially in pathologic states such as colitis (Lakshmi and Joshi, 2005; Sugiura et al., 2007; De Schepper et al., 2008; Christianson et al., 2009; Malin et al., 2009). In addition to P2X receptors, P2Y receptors are also involved in the initiation or modulation of nociception. A recent study by Hockley et al. (2016) showed that P2Y_1_ and P2Y_2_ receptor activation (i.e., by ADP and UTP) stimulate mouse and human visceral nociceptors suggesting the possible involvement of P2Y-dependent mechanisms in the generation of visceral pain in GI disorders.

### Future directions

Work done in the BON cell model on purinergic signaling will need to be verified in normal hEC cells from surgical specimens. A caveat is that EC cells are extremely sensitive to mechanical stimulation or any manipulations that alter the shape of the cells or deformation of the membrane in EC cells. Therefore, one of the unavoidable short-comings of enzymatic isolation, FACS sorting of EC cells is that such extreme isolation techniques, necessary to separate and purify hEC cells from their natural environment, will undoubtedly alter their responsiveness, and could lead to alterations in transcriptional regulation of receptors, channels and other proteins involved in the physiology of EC cells. This makes it more difficult to interpret data on EC cells from control and inflamed/IBD tissues. Therefore, a fluorescently tagged EC cell mouse model with either GFP (Schmidt et al., 2013) or CFP (Li et al., 2014) could provide a means to target single EC cells *in vitro* in their intact microenvironment, the mucosa lining the GI tract, by Ca^2+^ imaging, patch-clamp, electrochemical detection of 5-HT or purines to study modulation of 5-HT secretion.

Information is lacking on the role of Piezo, purinergic or other components of the mechanotransductive pathway in EC cells in gut motility reflexes. The technology is available to explore ways to engineer EC-cell specific KO mouse models for purinergic receptors (P2Y_1_, P2Y_12_, A_2B_) or Piezo 2 channels to study their role in the physiological regulation of mucosal gut reflexes or the involvement in the pathophysiology of GI disorders or inflammatory bowel diseases. Different molecular approaches can be taken in order to identify and sort out EC cells of the intestine *in vivo*. A Bacterial Artificial Chromosome (BAC) transgene approach uses long fragments of genomic DNA containing all the regulatory region of a specific gene to generate cell-specific transgenic animals. This approach has been used to successfully generate mouse lines faithfully mimicking the endogenous locus expression especially for the study of a specific neuronal population (Heintz, 2001; Yang and Gong, 2005). This technology can be explored further to generate EC-cell specific transgenic mouse models to study physiological regulation of specific purinergic or other targets linked to mechanotransduction in gut reflexes.

A novel revolutionary technology to effectively edit the mouse genome and generate new mouse lines in a fraction of the time required with the classical methods has recently become available to the mouse modeling community (Wang et al., 2013; Yang et al., 2013; Singh et al., 2015). This technology, called CRISPR (Clustered Regularly Interspaced Palindromic Repeats, which the Mouse Modeling Core at OSU has optimized) will allow us to create better models modifying and tagging genes in the endogenous context bypassing the need of extra pieces of DNA and the inherent issues associated with their forced expression.

Responses to purines (and perhaps Piezo 2 channels) are likely to be polarized, as has been shown for A_2B_ receptor activation of epithelial cells (reviewed by Cooke and Christofi, 2006). Therefore, studies that can discriminate between luminal and basolateral effects of purines on 5-HT secretion are essential to address the physiological and pathophysiological regulation of purines on 5-HT secretion and initiation of gut reflexes. It is worth pursuit, to carry out such studies in human mucosal biopsy from endoscopic examination, to study normal, IBD and IBS biopsy. The receptors and mechanisms involved can thus be investigated. Polarized responses could potentially have differential sensitivity to intestinal inflammation.

Patch-clamp analysis was used to evaluate the effects of the uridine neucleotide UTP on currents and membrane potential (V_m_) linked to mechanosensitivity and purinergic modulation—Findings to date indicate that UTP can modulate V_m_ and K_*V*_ channels in BON cells (Linan-Rico et al., 2014). Studies on ionic mechanisms of purinergic modulation of mechanosensitivity are worth pursuit. One possibility is that ATP release occurs as a result of activation of one or more mechanosensitive channels in EC cells, given that ATP release occurs in response to mechanical stimulation. Studies in normal EC cells are needed to confirm their presence and levels of expression in normal (or disease states), and involvement in mechanosensory transduction leading to 5-HT release. The Piezo 2 channel is a mechanogated channel identified in EC cells for mechanotransduction in secretomotor reflexes (Wang et al., 2016). Whether Piezo 2 activation (or other mechanogated channels) leads to ATP release in EC cells is a fundamental question.

There are known species differences in P2Y_4_, P2Y_11_, P2X_1_/P2X_2_, A_3_, and A_1_ receptors, and ligands that are suitable in the mouse (guinea pig or rat) may not necessarily work the same in human EC cells. Secondly, notable species differences in purinergic signaling between animals and humans (Cooke et al., 1999, 2004; Kennedy et al., 2000; Wunderlich et al., 2008; Liñán-Rico et al., 2015), make it necessary for studies to compare mouse and human EC, to identify suitable targets in mouse for detailed mechanistic studies and take advantage of genomic animal models, that could also be relevant to human EC physiology and pathophysiology.

In the physiological setting, extracellular ATP or UTP levels (or their metabolites adenosine, ADP, AMP, and UDP) are kept low (~10 nM) that may be sufficient to cause a transient increase in IP_3_-dependent Ca^2+^ signals (Koizumi et al., 2002). However, cellular injury, mechanical stress, inflammation or activation and degranulation of mast cells may increase levels of nucleotides to levels that are sufficient to evoke much larger, and more persistent global Ca^2+^ signals in cells (Osipchuk and Cahalan, 1992; Lazarowski et al., 1997, 2003) including EC cells. These aspects are worth investigation.

## Summary and conclusions

Our hypothesis for mechanotransduction in EC cells is illustrated in Figure 1. The focus of studies on mechanotransduction in EC cells studied in BON cells, other cell lines, mouse EC cells or most recently human EC cells refer to purinergic autocrine modulation of 5-HT release by ATP, UTP and adenosine for moment-to-moment fine-tune modulation of 5-HT release from EC cells. Release of these purines from surrounding epithelial cells contributes to paracrine stimulation of EC cells. Cell to cell communication in response to mechanical stress occurs via bilateral release of purines in polarized epithelia. Moreover, it is not known whether the same or different mechanosensory—mechanotransduction pathways, including those involved in releasing purines (ATP and UTP) operate in EC cells and surrounding epithelial cells during peristalsis. Furthermore, it is also not clear what physiological conditions favor autocrine (EC release of purines) or paracrine (epithelial release of purines) purinergic regulation. Both 5-HT and ATP can trigger intrinsic sensory reflexes, although to date, the most completely studied signaling pathway is 5-HT. Our unified hypothesis is that autocrine purinergic regulation provides local, moment-by-moment fine tune modulation of 5-HT release involved in intrinsic sensory gut reflexes, whereas paracrine purinergic regulation by surrounding epithelia provides more global regulation of 5-HT secretion and coordination of the movements of large portions of the gut. The mechanisms are not understood.

Release of 5-HT has a myriad of functions beyond intrinsic gut reflexes, including transmission of satiety signals, pain signals, induction of emesis, and the pathogenesis of intestinal inflammation (Figure 2). Abnormal 5-HT signaling occurs in IBD and GI disorders, but little is known about how it occurs in EC cells. Purinergic signaling is very sensitive to inflammation, and this is also the case in EC cells. 5-HT release is tightly regulated by purines, and it is therefore likely that purinergic mechanisms are linked to abnormal 5-HT secretion and hence signaling in inflamed gut. Beyond the EC cell, according to Burnstock's hypothesis of purinergic mechanosensory transduction of visceral pain, excess, bulk ATP release from surrounding epithelial cells activates subepithelial sensory nerves transmitting pain signals to the CNS via P2X3 receptor activation. The emerging picture is a complex regulation of intestinal reflexes by 5-HT and ATP with important interactions occurring between them in normal and inflamed diseases. Arguably, a better understanding of such interactions is a critical step in elucidating mechanisms of abnormal 5-HT signaling in disease states.

**Figure 3:**
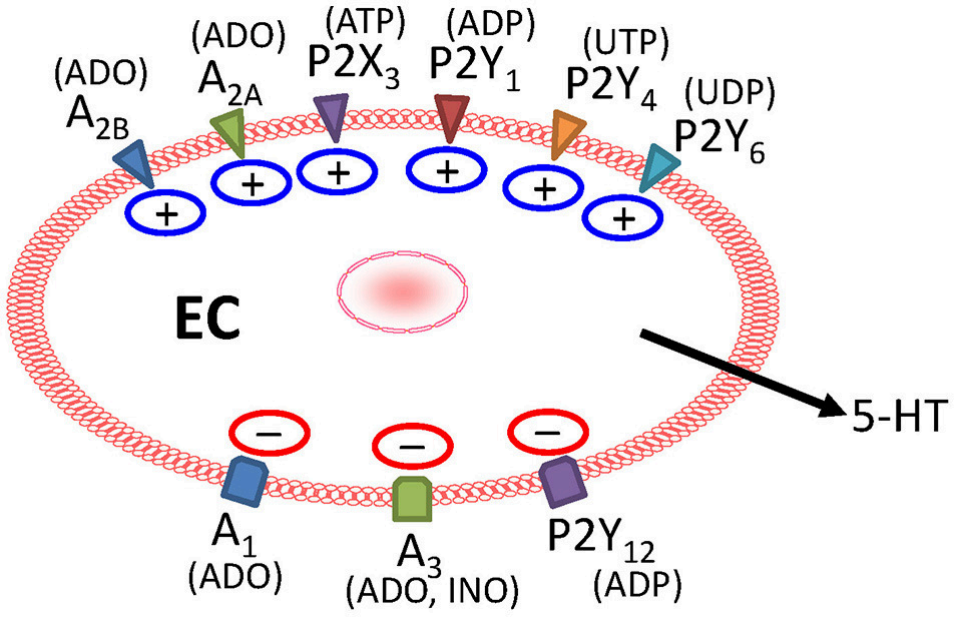
**Purinergic receptors provide dual modulation of mechanically—evoked 5-HT release in BON cells**. MS releases purines to act on stimulatory A_2_ (A_2*A*_ and A_2B_ receptors activated by ADO), ATP-gated P2X_3_ (activated by ATP), P2Y_1_ (activated by ADP), P2Y_4_ (activated by UTP), or P2Y_6_ (activated by UDP) receptors to stimulate 5-HT release. During MS, activation of inhibitory A_1_ (activated by ADO), A_3_ (activated by ADO/INO, inosine), or P2Y_12_ (ADP) receptors leads to attenuation of 5-HT release. Not all receptors are expressed on the same cells, and stimulatory P2Y_1_ and inhibitory P2Y_12_ receptors are expressed on different populations of EC cells.

Mechanotransduction is a fundamental physiological mechanism in EC cells, leading to 5-HT and ATP release, but there are still many unanswered questions. What is the mechanosensor(s)? What is the relationship between Piezo 2 channels and ATP release? Are other mechanogated channels involved? Can the GPCRs act as the mechanosensor? What is the physiological role of Piezo 2 channels and purinergic receptors in EC cells in gut motor reflexes and visceral sensation? What are the purinergic receptors linked to mechanotransduction in normal mouse and human EC cells compared to those in human BON cells (see Figure 3)? To what extend is altered purinergic signaling in EC cells responsible for abnormal 5-HT signaling in IBD and IBS?

Answering these questions raises the possibility that drugs targeting several distinct signaling pathways linked to mechanical stimulation, including Piezo 2 mechanogated channels, the P2Y_1, 4, 6_—Gαq/PLC/IP_3_-SERCA—Ca^2+^ signaling pathway, P2Y_12_—AC/cAMP signaling pathway, P2X—channels, or adenosine GPCRs (A_1_, A_2A_, A_2B_, and A_3_) could provide important novel targets for therapeutic interventions where improper mechanosensation of EC cells (and abnormal gut reflexes) can contribute to symptoms of diarrhea, constipation or visceral pain. Finally, off-target GI effects should be considered for drugs such as clopidogrel (Plavix) that irreversibly binds P2Y_12_ receptors (Berger, 2013) that could potentially disrupt motor functions.

## Author contributions

AL-R, FO-C, AB, SS, AZ-A, VC, and FC all contributed to writing the overall review article as well as contributed to concepts and ideas proposed. FC was responsible for overall organization, content, layout, and illustrations, integration of various sections provided by co-authors, production, and submission. AL-R contributed to sections on mechanosensitivity in BON(EC) cells, Ca^2+^signaling and 5-HT release, and P2Y1, P2Y12, and P2X3 receptors in human EC cells in normal and inflamed gut. She is primary author on some of the references cited. FO-C contributed to sections on ion channels and UTP signaling in BON(EC) cells. He co-authored some of the references cited in the review. AB wrote the section on Piezo 2 mechanogated channels illustrated in Figure 1, and contributed to editing of the overall manuscript. SS was responsible for internet searches to identify relevant publications on BON(EC) cells, and contributed to overall organization, writing and editing. AZ-A contributed to all aspects of the review article, internet searches for articles, development of concepts and hypotheses and editing the overall manuscript. VC co-wrote a portion of the section on Future Directions and contributed to those portions of the article that refer molecular signaling studies, CRISPER technology and BAC transgene technology.

### Conflict of interest statement

The authors declare that the research was conducted in the absence of any commercial or financial relationships that could be construed as a potential conflict of interest.

## Abbreviations

5-HT: 5-hydroxytryptamine
AC: adenylyl cyclase
CD: Crohn's Disease
CFP: cyan fluorescent protein
CgA: Chromagranin A
CNS: central nervous system
DRG: dorsal root ganglion
EC: enterochromaffin cells
ENS: enteric nervous system
FFA: free fatty acids
FRET: Fluorescence Resonance Energy Transfer
GFAP: glial fibrillary acidic protein
GFP: green fluorescence protein
GI: gastrointestinal
GPCRs: G protein coupled receptors
hEC: human enterochromaffin cells
IBD: Inflammatory Bowel Disease
IBS: Irritable Bowel Syndrome
KO: knock out
MS: mechanical stimulus
PKA: cAMP dependent protein kinase A
PLC: phospholipase C
Tph1: tryptophan hydroxylase 1
UC: Ulcerative Colitis
